# Cognition and Synaptic-Plasticity Related Changes in Aged Rats Supplemented with 8- and 10-Carbon Medium Chain Triglycerides

**DOI:** 10.1371/journal.pone.0160159

**Published:** 2016-08-12

**Authors:** Dongmei Wang, Ellen S. Mitchell

**Affiliations:** Nestle Institute of Health Sciences, Cognitive Health and Aging, EPFL Innovation Park, Building H, 1015, Lausanne, Switzerland; Universidade do Estado do Rio de Janeiro, BRAZIL

## Abstract

Brain glucose hypometabolism is a common feature of Alzheimer’s disease (AD). Previous studies have shown that cognition is improved by providing AD patients with an alternate energy source: ketones derived from either ketogenic diet or supplementation with medium chain triglycerides (MCT). Recently, data on the neuroprotective capacity of MCT-derived medium chain fatty acids (MCFA) suggest 8-carbon and 10-carbon MCFA may have cognition-enhancing properties which are not related to ketone production. We investigated the effect of 8 week treatment with MCT8, MCT10 or sunflower oil supplementation (5% by weight of chow diet) in 21 month old Wistar rats. Both MCT diets increased ketones plasma similarly compared to control diet, but MCT diets did not increase ketones in the brain. Treatment with MCT10, but not MCT8, significantly improved novel object recognition memory compared to control diet, while social recognition increased in both MCT groups. MCT8 and MCT10 diets decreased weight compared to control diet, where MCFA plasma levels were higher in MCT10 groups than in MCT8 groups. Both MCT diets increased IRS-1 (612) phosphorylation and decreased S6K phosphorylation (240/244) but only MCT10 increased Akt phosphorylation (473). MCT8 supplementation increased synaptophysin, but not PSD-95, in contrast MCT10 had no effect on either synaptic marker. Expression of Ube3a, which controls synaptic stability, was increased by both MCT diets. Cortex transcription via qPCR showed that immediate early genes related to synaptic plasticity (arc, plk3, junb, egr2, nr4a1) were downregulated by both MCT diets while MCT8 additionally down-regulated fosb and egr1 but upregulated grin1 and gba2. These results demonstrate that treatment of 8- and 10-carbon length MCTs in aged rats have slight differential effects on synaptic stability, protein synthesis and behavior that may be independent of brain ketone levels.

## Introduction

Octanoic acid, and decanoic acid, 8-carbon and 10-carbon medium chain fatty acids (MCFA), respectively, are found as medium chain triglycerides (MCT) in foods such as coconut oil and goat milk. Unlike longer chain fatty acids such as palmitic acid, MCFA are able to quickly enter the bloodstream and exist as free fatty acids which freely pass through cell membranes. Upon entry into the portal vein MCFA are converted into ketones (beta-hydroxybutyrate, acetate and acetoacetate), which can be taken up into the brain via MCT1 transporters [1]. Ketones act as substrates for production of acetyl-CoA, thus they can serve as alternative energy sources when glucose is low [2]. This glycolysis bypass property of ketones is especially important for neurons, which use glucose or ketones as primary energy sources for production of ATP. Alzheimer’s disease (AD) patients have been shown to exhibit decreases in brain glucose metabolism and glycolytic enzymes, thus ketones may be useful as an adjuvant fuel [3]. Recent research has shown that ketones derived from medium chain fatty acids improve cognition in diabetic and AD patients, and attenuate neurodegeneration in an ALS mouse model [4].

Although a majority of MCFA ingested are converted into ketones, ADME studies have shown that MCFA themselves can enter the brain and have diverse effects on neuronal function [5,6]. For example, medium chain fatty acids activate free fatty acids sensing G-protein coupled receptors such as GPR40 and GPR84, which are highly expressed in the brain and have roles in glucose regulation and inflammation [7]. Recently medium chain fatty acids have been shown to modulate mitochondrial enzyme function in a neuroblastoma cell line [8]. Specifically a 6-day administration of decanoic acid increased citrate synthase, Complex I and catalase activity while octanoic acid had no effect. Interestingly, decanoic acid has been shown to be a ligand for PPAR gamma receptors, while octanoic acid has no activity [9]. Meanwhile, octanoic acid has been almost exclusively used to in human and animal studies assessing cognition, due to its purported superior ketogenic potential.

It is unknown if decanoic acid triglyceride (MCT10) also has effects on cognition, since no published studies have assessed MCT10 for effects on age-related cognitive decline. Moreover, it is currently unknown if MCFA such as octanoic acid or decanoic acid, which are released after MCT consumption, have differential effects on metabolic function of neuronal cells in vivo.

In the present study MCT10 and MCT8 treatments were fed to aged rats for 8 weeks, and their cognitive performance was compared to aged rats given a sunflower oil supplemented diet.

## Methods

### Animals, treatment, measurement of ketones and MCFA

All animal care and procedures were approved by the Nestle Institutional Ethic Committee for Animal Care and the Swiss Federal Food Safety and Veterinary Office (FSVO). Rats were deeply anesthetized via isoflurane, and decapitated. Thirty-six male Wistar rats, 21 months of age, were ordered from Janvier Labs (France) and acclimated for 7 days to the facility. During this time, they were kept in pair-housed cages. After the acclimatization period, they were randomized into three groups of 12 animals according to cognition and body weight. Baseline cognition was tested via social recognition (procedure described below). Rats were single-housed in standard cages with free access to food and water, and were kept at a 12 hour light cycle period, at a temperature between 20–24°C, and a relative humidity between 50–60%. Rats were given a diet of 5% medium chain triglycerides: octanoic triglyceride (MCT8), decanoic triglyceride (MCT10) or control diet (5% sunflower oil) via specially formulated animal chow for 8 weeks. Sunflower oil was added to the control diet in order to maintain an equivalent calorie and lipid content to that of the MCT-supplemented diets. Rats were weighed every week to ensure weight maintenance. Plasma was collected at sacrifice via heparinized tubes, and was analyzed for ketones via colorimetric kits (Cayman) and medium chain fatty acids, octanoic and decanoic acid, were analysed via GC/MS [10]. Brains were extracted and snap-frozen in liquid nitrogen. Analysis of brain tissue concentrations of beta-hydroxybutyrate (BHB) were performed by GC/MS (see Scherer). Samples were prepared via liquid/liquid extraction of 50 μl supernatant (pH 2), and extract derivatization with MSTFA (silylation). GC/MS analysis was performed using an internal standard 13C2-BHB, m/z 233 (BHB), m/z 118 (13C2-BHB) using a calibration of 2–24 μg/ml (water); LOQ: 2 μg/ml, LOD: 0.7 μg/ml.

### Behavior testing

#### Social recognition

Rats were tested for behavioral effects of MCT via a social interaction test 3 days before sacrifice. This test involves the introduction of an unfamiliar juvenile rat into the home-cage of the test rat for 4 minutes. The amount of touching, sniffing and general exploration of the juvenile rat was recorded, and the total time period of social interaction were used as indicators of sociality and anxiety. After 1 hr the previously explored juvenile and a new juvenile were introduced into the home cage for 4 minutes. If a rat failed explore the juvenile for more than 30 seconds during the first session it was not included in the final analysis. Social recognition was calculated by the amount of time spent exploring the new juvenile over the total amount of time spent exploring both juveniles (JNEW/(JNEW+JOLD)).

#### Open field and object recognition

The novel object recognition test provides both a measure of anxiety, locomotion (open field), and exploratory behavior (novel object). Animals were placed in the intermediate zone of a square open field (100X100 cm2) with a low-level lighting to avoid inducing state anxiety (25 Lux) and allowed to freely explore the arena for 5 minutes. In this test, the distance traveled is a valid measure of natural locomotor activity. The time spent in the center of the arena in the first 5 minutes is a measure of anxiety. During this time, the animal’s behavior was recorded and automatically tracked using Ethovision tracking software, and after each session the arena is cleaned with 70% ethanol. The following day, two identical objects (small, colored cylindrical or rectangular shapes) were placed in the center of the arena and the animal was allowed to explore the objects for 10 minutes or until the total duration of object exploration is 30 seconds. If a rat failed to explore the objects for at least 30 seconds it was not included in the final analysis. After 1.5 hr the rat was placed in the field with the previous object and a new object for 5 min. The amount of time spent in the center with the objects present during the first session is a valid measure of exploratory activity and the amount of time spent exploring the novel object vs. total time exploring both objects in the second session is an indicator of memory ability.

### Protein extraction

Rat brain tissue samples were homogenized in a buffer containing NaCl 1M, EDTA 0.5M,EGTA 0.5M, NaF 0.5M, Na4O7P4 0.2M, Na3VO4 0.5M, Triton-100, SDS 10%, deixycholate 5%, PMSF 200mM and protease inhibitor cocktail (Roche). The protein was obtained after centrifugation of the homogenate at 10000 g for 10 min at 4°C. The protein concentration was determined by Bradford method.

### IRS-1, IGF-1, GDNF, and VEGF ELISA

Proteins extracted from rat forebrain tissue were used to detect glial cell-line-derived neurotrophic factor (GDNF), insulin growth factor 1 (IGF-1), total and phosphorylated ser(307) insulin receptor substrate 1 (IRS-1) and vascular endothelial growth factor (VEGF). The GDNF Emax^®^ ImmunoAssay System from Promega (Cat. # G7621) was used to detect GDNF levels. Briefly, 96-well plates from Sigma (Nunc-Immuno^™^ MaxiSorp^™^, Cat. # 439454) were coated with anti-GDNF Monoclonal antibody provided in the kit at 4°C overnight. After blocking for 1 hour at room temperature with Block & Samples buffer, 30μg of protein sample from each rat was added and incubated at room temperature for 6 hours. Then the plates were washed 5 times with wash buffer, followed by adding anti-Human GDNF pAb and incubated at 4°C overnight. After washing, a HRP Conjugated Anti-Chicken IgY was added to each well and incubated at room temperature for 2 hours. Following wash step, 100μl TMB One Solution was added to each well and incubated for 30 min at room temperature. The coloration was stopped by adding 100μl of 1N hydrochloric acid, and the absorbance at 450nm was measured on plate reader (FlexStation3, Molecular Devices) within 30 minute. ELISA kits from Invitrogen and R&D Systems were used to measure IRS-1 (Cat. #KHO0521), VEGF (Cat. #KMG0111) and IGF-1 levels following the manufacturer’s instructions. For each brain sample, 30μg protein was used, and all samples were detected in duplicate.

### Western blots

NuPAGE Novex Midi Gels (4–12%) from Life Technologies were used for protein separation. The following antibodies were used: Anti-PSD95 antibody (Abcam, Cat. #ab18258), Synaptophysin (D35E4) XP^®^ Rabbit mAb (Cell Signaling, Cat. #5461), Complex II Subunit Monoclonal Antibody (clone 21A11AE7, Life Technology, #439454), Phospho-p70 S6 Kinase (Thr421/Ser424) (Cell Signaling, #9204), S6K(p70) (Cell Signaling, #2217), Phospho-Akt (Ser473) Antibody (Cell Signaling, Cat. #9271s), Akt (pan) (11E7) Rabbit mAb (Cell Signaling, Cat. #4685s), Ube3a (Abgent, #346954), β-Tubulin Antibody (TUB 2.1, Santa Cruz, Cat. #sc-58886) and β-Actin antibody (Sigma, Cat. #A1978). After incubating with corresponding infrared fluorescent secondary antibodies from Li_COR Biosciences (#926–32213 IRDye^®^ 800CW Donkey anti-Rabbit IgG, and #926–68072 IRDye^®^ 680RD Donkey anti-Mouse IgG), the signal of specific protein was detected and quantified on the Odyssey CLx imager (LI-COR).

### Gene expression detection by PCR

Total RNA was extracted and purified with the RNAdvance tissue kit (Agencourt, Beverly, MA, USA). The quality of RNA samples was checked by using the Fragment Analyzer (Advanced Analytical Technologies, Inc, Ames, IA, USA). Reverse transcription was performed using PrimeScript Reagent kit from Katara Clontech (Cat. #RR037A) following the manufacturer’s instructions using 500 ng of each RNA sample. After adding LightCycler^®^ 1536 DNA Green Master (Roche, Cat. #5573092001), gene expression was quantified on a Roche LightCycler 480. Primers for the following genes were used for the PCR reaction: Arc, Egr1, Egr2, Fosb, Srf, nr4a1, Plk3, Junb, Grin1, Gba2, and beta-tubulin was used as an endogenous control.

Rat-Egr1-F: aacaaccctacgagcacctg; Rat-Egr1-R: aaaggggttcaggccacaaa;

Rat-Fosb-F: gccttcaactagcacaagcac; Rat-Fosb-R: ctgatccgtttccgcctgg;

Rat-Srf-F: atgcagtgatgtatgccccc; Rat-Srf-R: cagccatctggtgaagctga;

Rat-Nr4a1-F: gcatggtgaaggaagttgtcc; Rat-Nr4a1-R: aaaattgctgcacgtcaccg;

Rat-Egr2-F: aaacggcttctctggcactc; Rat-Egr2-R: ttgatcatgccatctccagcc;

Rat-Junb-F: gtttacatggcccccttcca; Rat-Junb-R: agtatccccacaggctgagt;

Rat-Arc-F: acagacacagcagatccagc; Rat-Arc-R: tgagtcatggagccgaagtc;

Rat-Gba2-F: ctaccctgcatgttgtccgt; Rat-Gba2-R: tcagctgtccggaaaccttc;

Rat-Grin1-F: cttcagtccctttggccgat; Rat-Grin1-R: agttggcagtgtaggaagcc,

Rat-Actin-F: gtcgtaccactggcattgtg; Rat-Actin-R: ctctcagctgtggtggtgaa

Rat-Plk3-F: gcaagcagtggagatggatt; Rat-Plk3-R: ggacagctgatagccaaagc

### Statistical analysis

One-way ANOVA were used for analyses of behavior, protein and mRNA expression. Weight change was analysed via repeated measured ANOVA. If significant differences were found between groups then the post-hoc tests used were Tukey, for comparisons to the control group or Bonferroni comparisons between the MCT groups. All data are presented as the mean (± SEM).

## Results

### Weight, plasma ketones and medium chain fatty acids

**Fig 1:**
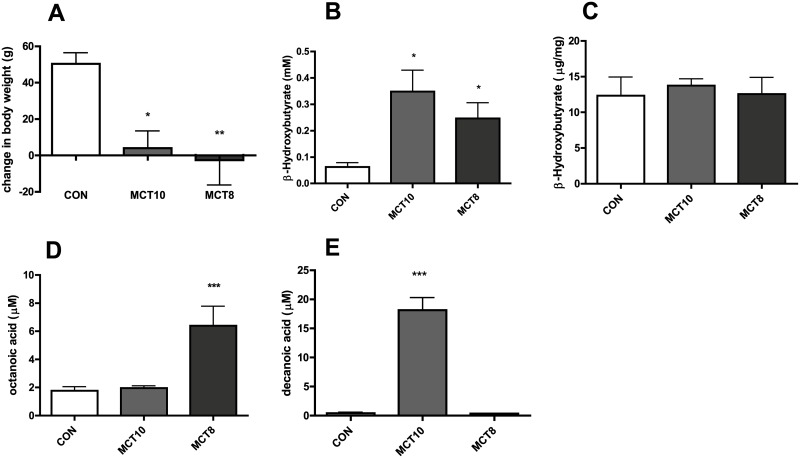
MCT8 and MCT10 groups maintained weight while exhibiting higher levels of ketones and MCFA compared to the control group. (A) percentage change in weight, *p<0.05, **p = 0.01 vs. control, n = 9–12 per group. (B) plasma beta-hydroxybutyrate (mM), *p<0.05, n = 7–8 per group. (C) brain hydroxybutyrate (μg/ml), n = 3–4 per group. (D) plasma octanoic acid (μM),***p<0.001, n = 8 per group. (E) plasma decanoic acid (μM), ***, p<0.001, n = 8 per group; All data are mean + SEM.

The diet appeared to be well-tolerated by all groups, however, due to the extreme age of the animals 3 animals died in the last week of the intervention: 1 mortality in the control group and 2 mortalities in the MCT8 group. These animals were not included in the behavior, post-mortem or blood analyses. There was no significant effect of MCT treatments on food intake (average intake per day in grams: CON, 23.3± 2.4; MCT8, 21.6± 1.4; MCT10 22.1± 1.8). Despite no difference in food intake, both MCT treatments maintained similar body weight throughout the intervention while the sunflower oil supplemented group gained weight (Fig 1A). Plasma beta-hydroxybutyrate (BHB) as measured by colorimetric kits increased in the MCT10- and MCT8-treated group compared to controls (Fig 1B), yet brain BHB as measured by GC-MS were similar in all groups (Fig 1C). Measurement of plasma octanoic acid in MCT8-treated groups averaged at 6.4 ± 1.4 μM (Fig 1D) while average levels of plasma decanoic acid for the MCT10 group was 18.2 ± 2.1 μM (Fig 1E).

### Cognition

**Fig 2:**
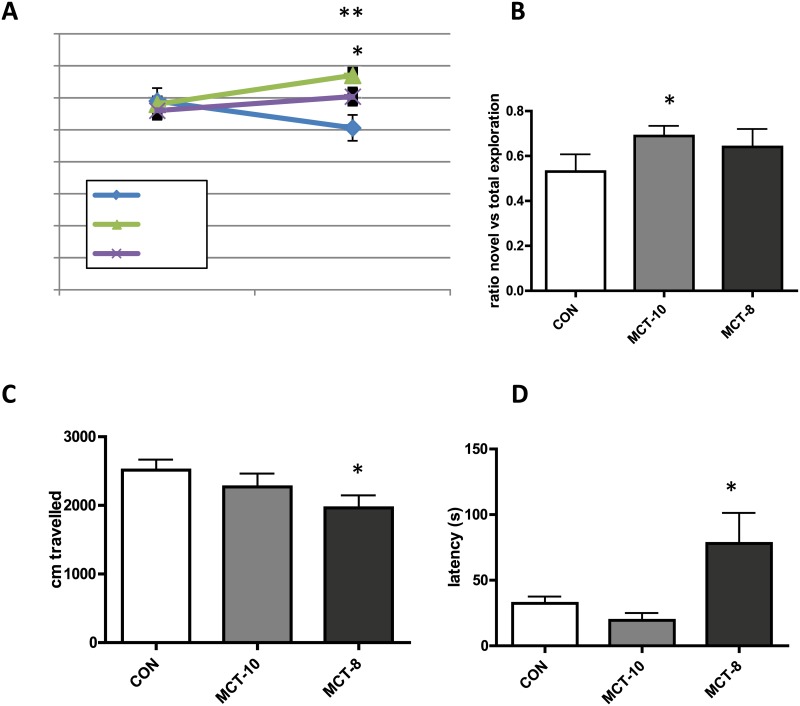
Cognitive performance was increased in MCT groups compared to control diet by one way ANOVA analysis. (A) social recognition at baseline and 8 weeks after chronic MCT treatment.**p<0.01; *p<0.05 vs. control. n = 10 per group. (B) novel object recognition after 8 wk chronic MCT treatment, * p<0.05 vs. control; n = 9 per group. (C) distance travelled in an open field. * p = <0.05 vs control. (D) latency to touch a novel object in arena center, * p<0.05 vs control; All data are mean + SEM.

Rats at advanced ages exhibit wide variations in cognitive ability. To ensure that all groups had similar average cognition, a social recognition test was performed before diet initiation, and average social discrimination was slightly above chance level for a majority of the rats (Fig 2A). Rats were then randomized into treatment groups according to cognition, and eight weeks after the treatment initiation social recognition was tested again. While there were no group differences in initial social exploration of a juvenile (data not shown), rats given MCT10 or MCT8 supplemented diet had a social discrimination ratios of novel vs. familiar juveniles significantly higher than the control group (Fig 2A).

Novel object recognition (NOR) measures long term episodic memory in a manner similar to social recognition. NOR was performed one day before sacrifice and results indicate a significant improvement of novel object recognition in the MCT10 rats compared to control, but not the MCT8 rats (Fig 2B).

### Anxiety and exploratory behavior

In the open field test there was no treatment effect observed in latency to enter the center area, or in total time spent in the center (data not shown). There was a significant decrease in total average distance covered in the MCT8 treated rats compared to control rats that was not observed in MCT10 rats (Fig 2C, F(2,32) = 7.40, p<0.05) and in terms of initial exploration of the novel object rats given MCT8 showed higher latency to explore an object placed in the arena (Fig 2D, F(2,32) = 6.40, p<0.05).

### Protein expression and activation

**Fig 3:**
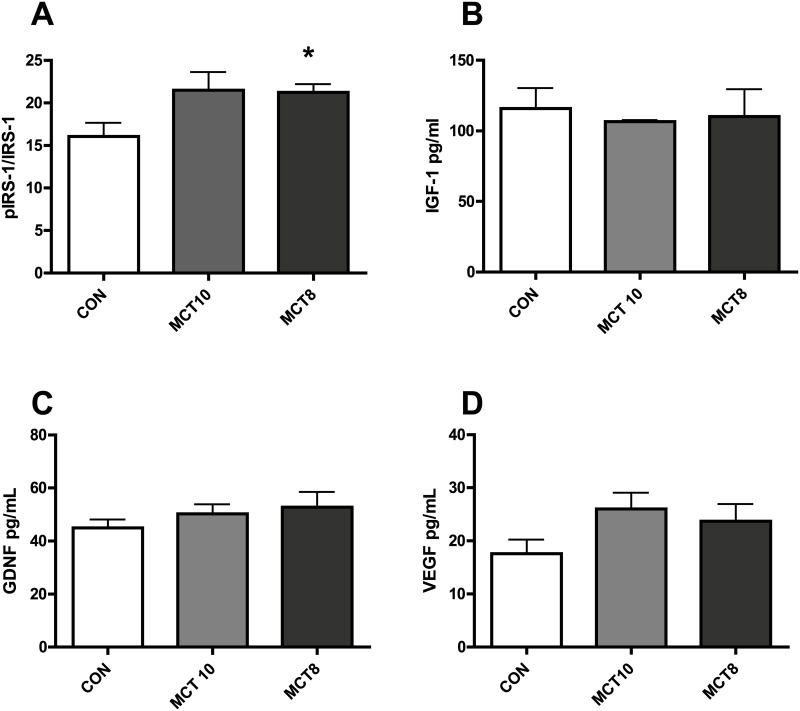
Insulin signaling protein phosphorylation and growth factors. (A) IRS-1 phosphorylation ser(307) *, p<0.05 compared to control (CON). (B) hippocampal IGF-1 (C) hippocampal GDNF. (D) hippocampal VEGF, p = 0.06, MCT10 compared to control. All data are mean + SEM. n = 5–6 per group.

Insulin signaling proteins and phosphorylation may be interesting targets for neuroprotection in Alzheimer’s related dementias [11,12]. MCT are known to affect insulin signaling pathways in the periphery [13] but have not been investigated in brain tissues. Protein expression analysis indicated that IRS-1 ser(307) phosphorylation was higher in MCT8-treated forebrain tissue (and tending towards higher expression with MCT10 diet) (Fig 3A, F(2,16) = 3.88), while no changes were seen in total IRS-1 (data not shown). Changes in hippocampal growth factors are often observed after cognition-enhancing treatments [14]. Thus it was hypothesized that MCT effects on cognition were due to increased hippocampal growth factor expression. Growth factors known to increase IRS-1 phosphorylation or synaptic plasticity were measured, but no significant differences were seen in forebrain VEGF, GDNF or IGF-1 expression (Fig 3B, 3C and 3D).

**Fig 4:**
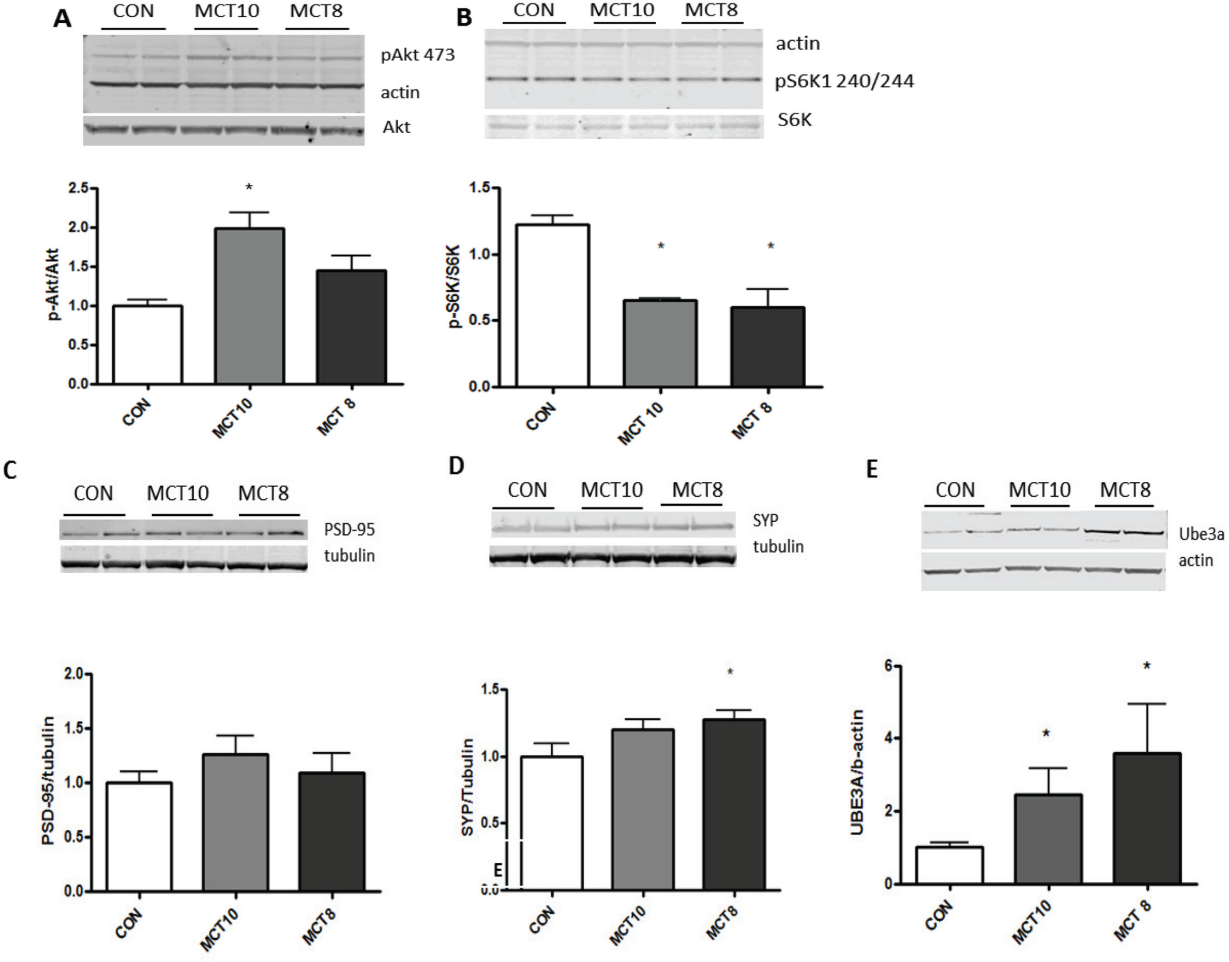
synapse-associated proteins and growth-related pathways. (A) S6K(p70) phosphorylation (pS 240/244) F(2,8) = 10.8 *, p<0.05 compared to control. (B) Akt phsophorylation (pS473) F(2,8) = 8.58 *, p<0.05 compared to control (CON). (C) PSD-95 protein expression. (D) synaptophysin (SYP) protein expression F(2,8) = 3.29 *, p<0.05 compared to control (CON). (E) Ube3a protein expression F(2,8) = 4.891 *, p<0.05 compared to control. All data are mean + SEM. n = 6 per group.

Serine 307 phosphorylation of IRS-1 attenuates insulin signaling, including Akt phosphorylation [15]. Surprisingly hippocampal Akt phosphorylation ser(473) was significantly elevated in the MCT10-treated group, but not in the MCT8 group as compared to control (Fig 4B). Phosphorylation of ribosomal protein S6 kinase (S6K) is a key signal for protein synthesis and synaptic growth and is activated through Akt [16]. Both MCT8 and MCT10 diets decreased S6 kinase serine 420/424 phosphorylation compared to control (Fig 4A). Akt phosphorylation has also been associated with increased tau phosphorylation, and so hippocampal tau (pS181) was examined, but no difference was detected between any groups (data not shown).

Cortical synaptic markers decrease during aging and these losses correlate to worsening cognition [17,18]. Synaptic protein expression in forebrain was measured via PSD-95, a post-synaptic marker, was not significantly different in any of the groups (Fig 4C). Synaptophysin, a pre-synaptic protein, was upregulated in the cortex of MCT8-treated rats, but not significantly changed in MCT10 rats compared to control (Fig 4D). Ube3a protein expression decreases during aging, and is a key regulator of synaptic stabilization via its ubiquitination of Arc [19]. Cortical Ube3a was increased by both MCT diets (Fig 4E).

### Transcript expression

**Fig 5:**
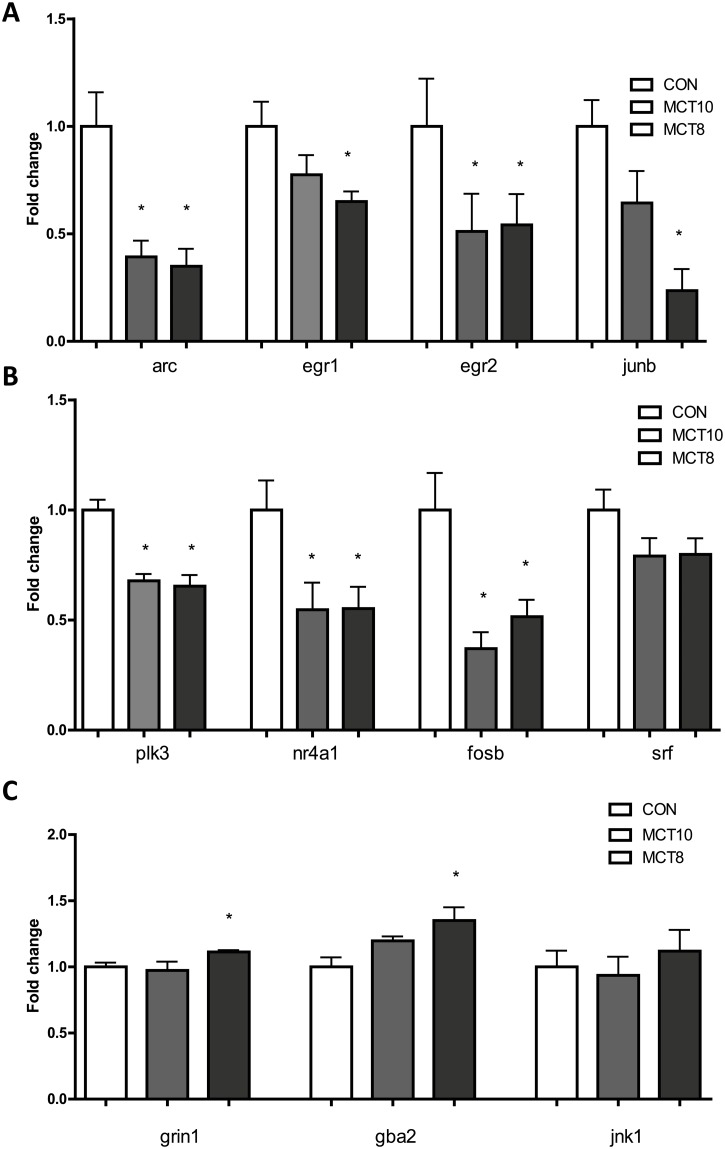
Relative mRNA expression in prefrontal cortex of MCT-treated rats: transcription factors associated with plasticity. (A) arc, egr1, egr2, junb. (B) plk3, nr4a1,fosb, srf. (C) grin1, gba2, jnk1. *, p<0.05. All data are mean + SEM. n = 6 per group.

Genes transcribing synaptic plasticity and immediate early genes were assessed in cortical samples. Transcription-related genes arc, egr1, egr2, junb, fosb, plk3, srf and nr4a1 were examined, and genes related to synaptic transmission: grin1, jnk1 (mapk8), and gba2 were also measured. A majority of immediate early genes measured were downregulated by both MCT treatments, where MCT8 treatment showed a tendency to decrease IEG transcripts to a greater degree (Fig 5A). MCT8 diet also increased selected markers of synaptic plasticity grin1 and gba2 mRNA transcription but not mapk8 expression (Fig 5B).

## Discussion

Medium chain triglycerides are currently prescribed for mild to moderate Alzheimer’s disease (AD) patients, and one clinical study has shown that MCT8 improves memory in APOE4-negative patients [20]. Several studies have demonstrated glucose hypometabolism in AD brains, and AD subjects with low cerebral metabolic use of glucose appear to compensate via higher brain ketone metabolism than age-matched controls [21,22]. While MCT-derived ketones are undoubtedly impacting brain metabolism, less is known about the individual effects of MCT-derived MCFA, octanoic acid and decanoic acid, which are readily available to the brain [5,23].

Both MCT diets were accepted by the rats, and although all the groups ate the same amount of chow, the MCT groups maintained weight while the sunflower oil group gained weight. This weight loss effect was likely due to immediate metabolism of MCT into ketones, which were then used as energy substrates in muscles and organs. Plasma ketones displayed a tendency of higher levels in MCT10 rats compared to MCT8 rats, but this was not significant. Previous MCT pharmacokinetic studies reported higher levels of ketones 3–6 hr after MCT8 gavage than MCT10, where MCT10 appeared to have a lower rate of metabolism into ketones. Our results do not support these findings, which only examined short term treatment [24,25]. However, in studies with humans demonstrate that prolonged feeding of MCT gradually increases plasma ketones over several weeks, suggesting that the rate of MCFA metabolism from MCT is saturated with chronic feeding [26].

Cognition has been shown to improve after octanoic triglyceride diet in aged humans and animals [2,27,28], yet no studies have compared specific species of MCTs in terms of cognition. The social recognition test measures olfactory episodic memory, involving the cortex and hippocampus [29]. This test robustly measures olfactory memory consolidation of juveniles and is highly sensitive to aging and nutritional interventions [18,30]. Control rats in the current study performed slightly above chance at baseline and after 8 weeks average ratios of recognition remained at chance level, indicating no recognition of familiar vs unfamiliar juveniles after 2 hours. MCT8 and MCT10 treated rats’ memory performance improved from baseline and, although there was no statistical difference between the two MCT treatments, MCT10 rats had greater trajectories of recognition indices in the social recognition test. In the novel object recognition test, MCT10 rats performed significantly better than control rats while MCT8 rats did not. Moreover, compared to control rats, MCT8 rats displayed less locomotor activity in an open field test, and had greater latency to explore an object placed in an open field, indicating lower motivation and novelty-induced exploration. MCT10 rats did not differ from control rats in these behavioral indicators. Taken together these results indicate a possible difference in behavioral effects due to MCT species, however, these results must be replicated and extended with tests assessing additional cognitive indices.

Synapse formation and maintenance in the hippocampus and cortex have long been associated with better cognition; thus two synaptic markers, PSD-95 and synaptophysin, were examined. While PSD-95, a post-synaptic marker, showed no difference between the groups, synaptophysin, located in pre-synaptic boutons, was increased in the MCT8 group compared to the control group. Interestingly, growth factors important for synaptic plasticity, VEGF, GDNF and IGF-1, were unchanged compared to controls. The IRS-1/Akt pathway has been shown to regulate neuronal growth via mTOR and p70S6K and in fact may direct neuronal fate towards apoptosis or growth, depending on intrinsic factors [31]. IRS-1 phosphorylation was increased by MCT8 diet while Akt phosphorylation was increased only in MCT10 rats, indicating differential activities related to MCFA carbon number. However, phosphorylation of p70S6K, a key regulator of protein synthesis and plasticity downstream of IRS-1 and Akt [32], was down-regulated by both MCT diets.

These findings suggest that, while octanoic and decanoic acid may activate IRS-1/PI3K/Akt signaling pathways to differential degrees, the net effect on protein synthesis regulation via S6K is similar. This may be due to direct effects of MCFA on neuronal receptors. For example, decanoic acid activates PPAR-γ receptors while octanoic acid metabolites, 2- and 3-hydroxyoctanoic acid, are HCA3 (GPR109B) agonists [9,33]. Interventions with PPAR-γ agonists such as rosiglitazone or pioglitazone have been shown to prevent apoptosis and mitochondrial dysfunction via increased neuronal Akt/PKB phosphorylation [34,35]. The mTORC1 complex is the major downstream target of Akt, and mTORC1 drives S6K phosphorylation, leading to subsequent protein translation and cell growth [36]. MCT10 increased Akt phosphorylation but unexpectedly decreased S6K phosphorylation, which suggests that other pathways are inhibiting S6K phosphorylation. Conversely, while MCT8 diet had no effect on Akt phosphorylation, S6K phosphorylation was decreased to the same extent as MCT10 diet. Ketogenesis has been shown to downregulate mTOR and S6K (13) and so it may be possible that S6K inhibition is due to raised ketones while Akt phosphorylation was due to MCT10-mediated PPAR-γ activity. Lastly, Ube3a, a key regulator of synaptic protein autophagy, was increased by both MCT diets. Ube3a, a binding component of the ubiquitin/proteasomal complex, has roles in transcription factor degradation and synaptic stabilization [37]. Decreased Ube3a expression, via genetic or pharmacological manipulation, causes cognitive dysfunction via loss of synapses [38], and thus MCT consumption may be enhancing cognition via synaptic maintenance.

Genes important for synaptic activity were also measured via RT-PCR in the prefrontal cortices of treated rats. Interestingly, a majority of IEGs essential for long term potentiation and memory consolidation were decreased in MCT treated rats, including arc, egr1, egr2, nr4a1, fosb, plk3 and junb. Arc generally measures how well a memory has been consolidated when it is quantified directly after a cognition test [37]. However, high basal Arc may indicate spontaneous activity of cells unrelated to specific memory formation (36). Basal Arc is upregulated in aged rats with poor memory while aged rats with unimpaired memory show similar low Arc expression to young rats [39]. Thus Arc is controlled homeostatically, i.e. induced during memory entrainment and then efficiently turned off to maintain the memory. Interestingly, Fletcher and colleagues showed that aged-impaired rats exhibited decreased hippocampal Ube3a protein expression and decreased expression correlated to higher Arc levels, suggesting that loss of Ube3a expression during aging may be contributing to constitutively active Arc, and thus poorer cognition.

Egr1 (NGFI-A), Egr2, FosB, and JunB belong to an early response family of transcription factors, where Egr1 is the most well-known for its role in synaptic plasticity, including regulation of Arc [40]. The above remaining transcription factors mediate synaptic renovation following long term potentiation (LTP) and are also tightly controlled. Accordingly, over-activation and chronic expression of plasticity-related IEG such as Egr1 indicates disrupted LTP mechanisms [41]. Junb, Nr4a1 (Nurr77) and Fosb, when transcribed directly after a memory task, are correlated with more efficient synaptic plasticity via LTD [42]. However, less is known about basal transcription of such genes during aging. Similar to the discussion above, inappropriate transcription of junb or nr4a1 may indicate dysregulated control of synaptic growth. Tightly controlled synaptic synthesis and autophagy of these transcription factors are necessary for memory consolidation and maintenance of memory [43]. Indeed, human and animal studies have shown that brain regions with higher rates of synaptic turnover are more vulnerable to amyloid plaque build-up and degeneration [44–46].

### Conclusion

This study compared the cognitive and physiological effects of two medium chain triglyceride diets in aged rats, with the aim of assessing differential effects. Both MCT diets improved cognitive performance and while the MCT10 diet tended to improve social recognition compared to the MCT8 diet, the effect did not reach significance. Slight differences were found between the two diets in terms of markers of synaptic, metabolic and transcriptional activity. MCT8 diet showed a tendency toward stronger modulation of specific transcription factors and synaptic markers than MCT10 diet, while MCT10 diet enhanced Akt phosphorylation compared to controls. However, further studies are required to ascertain if these putative effects indicate specific MCT species for cognitive decline treatment.

## Supporting Information

S1 Data BHB, MCFA and weight measurements raw data. (PZFX)

S2 Data Latency to explore object and behavior raw data. (XLS)

S3 Data Changes in growth factors and insulin signaling proteins. (PZF)

S4 Data Western blot raw data of synaptic proteins. (PZF)

S5 Data Prefrontal cortex gene expression: mRNA relative data of arc, agr1, egr2, junb. (PZFX)

S6 Data Prefrontal cortex gene expression: mRNA relative data of plk3, nr4a1, fosb, and srf. (PZFX)

S7 Data Prefrontal cortex gene expression: mRNA relative data of grin1, gba2, and jnk1. (PZFX)
